# In vitro and in vivo delivery of a sustained release nanocarrier-based formulation of an MRTF/SRF inhibitor in conjunctival fibrosis

**DOI:** 10.1186/s12951-018-0425-3

**Published:** 2018-11-27

**Authors:** Aristides D. Tagalakis, Shivam Madaan, Scott D. Larsen, Richard R. Neubig, Peng T. Khaw, Ian Rodrigues, Saurabh Goyal, Kin Sheng Lim, Cynthia Yu-Wai-Man

**Affiliations:** 1 Department of Ophthalmology, King’s College London, Westminster Bridge Road, London, SE1 7EH UK; 2 UCL Institute of Ophthalmology, 11-43 Bath Street, London, EC1V 9EL UK; 3 Department of Biology, Edge Hill University, Ormskirk, L39 4QP UK; 4 UCL School of Pharmacy, London, WC1N 1AX UK; 5 Vahlteich Medicinal Chemistry Core, College of Pharmacy, University of Michigan, Ann Arbor, MI USA; 6 Department of Pharmacology and Toxicology, Michigan State University, East Lansing, MI USA

**Keywords:** Nanocarrier, Sustained release, Inhibitor, Glaucoma, Fibrosis

## Abstract

**Background:**

Sustained drug delivery is a large unmet clinical need in glaucoma. Here, we incorporated a Myocardin-Related Transcription Factor/Serum Response Factor inhibitor, CCG-222740, into slow release large unilamellar vesicles derived from the liposomes DOTMA (1,2-di-O-octadecenyl-3-trimethylammonium propane) and DOPC (1,2-dioleoyl-*sn*-glycero-3-phosphocholine), and tested their effects in vitro and in vivo.

**Results:**

The vesicles were spherical particles of around 130 nm and were strongly cationic. A large amount of inhibitor could be incorporated into the vesicles. We showed that the nanocarrier CCG-222740 formulation gradually released the inhibitor over 14 days using high performance liquid chromatography. Nanocarrier CCG-222740 significantly decreased *ACTA2* gene expression and was not cytotoxic in human conjunctival fibroblasts. In vivo, nanocarrier CCG-222740 doubled the bleb survival from 11.0 ± 0.6 days to 22.0 ± 1.3 days (*p* = 0.001), decreased conjunctival scarring and did not have any local or systemic adverse effects in a rabbit model of glaucoma filtration surgery.

**Conclusions:**

Our study demonstrates proof-of-concept that a nanocarrier-based formulation efficiently achieves a sustained release of a Myocardin-Related Transcription Factor/Serum Response Factor inhibitor and prevents conjunctival fibrosis in an established rabbit model of glaucoma filtration surgery.

## Background

Drug delivery and duration of action remain major hurdles in the development of new anti-scarring therapies in the eye. Potential slow-release formulations, such as hydrogels [1], ocular tablets [2, 3], microspheres [4], microfilms [5], or ocular implants [6], are being developed for sustained drug delivery to the conjunctiva. Glaucoma is the leading cause of irreversible blindness in the world, currently affecting over 64 million people worldwide and its prevalence is estimated to rise to 76 million by 2020 and 112 million by 2040 [7]. Glaucoma filtration surgery (GFS) is the mainstay of surgical treatment but is often complicated by post-surgical fibrosis, which is the principal cause of blockage of aqueous flow at the drainage site leading to failure of surgery [8].

Serum response factor (SRF) is a ubiquitous transcription factor that controls growth factor regulated immediate early genes like c-fos and actin, as well as muscle-specific genes [9–11]. SRF is also a master regulator of cytoskeletal gene expression [12, 13], including many genes involved in fibrosis [14–17]. The Myocardin-related Transcription Factor family, MRTF-A and MRTF-B, is one of the principal families of signal-regulated SRF cofactors. They are controlled by cytoskeletal dynamics, responding to variations in the cellular concentration of G-actin to which they bind through N-terminal RPEL motifs [18, 19].

We have previously shown that repeated injections of an MRTF/SRF inhibitor, CCG-222740, effectively prevented scar tissue formation in a rabbit model of experimental GFS [20]. Our hypothesis is that a nanocarrier-based formulation of CCG-222740 will achieve a sustained release of the drug from a single administration and have similar efficacy compared to repeated injections. In this study, we evaluated in vitro the efficacy and safety of a novel sustained release nanocarrier-based formulation of CCG-222740 in human conjunctival fibroblasts. We also tested the effects of the nanocarrier CCG-222740 formulation on bleb survival and conjunctival fibrosis using a rabbit in vivo model of experimental GFS.

## Methods

### Materials

DOTMA (1,2-di-*O*-octadecenyl-3-trimethylammonium propane) and DOPC (1,2-dioleoyl-*sn*-glycero-3-phosphocholine) were purchased from Avanti Polar Lipids (Alabama, USA). CCG-222740 inhibitor was synthesised and characterised in the Vahlteich Medicinal Chemistry Core at the University of Michigan. The inhibitor was > 95% pure by high performance liquid chromatography (HPLC). HPLC grade (> 99% purity) solvents, including acetronitrile, trifluoroacetic acid, chloroform and methanol, were bought from Sigma, UK. All glassware was sterilised by autoclaving and procedures were performed under aseptic conditions in a cell culture hood.

### Preparation of large unilamellar vesicles

Nanocarrier formulation of CCG-222740 was prepared by the thin film hydration technique as previously described [21]. Equal amounts (0.66 mg) of DOTMA (lipid concentration dissolved in chloroform = 25 mg/ml) and DOPC (lipid concentration dissolved in chloroform = 25 mg/ml) were added to CCG-222740 (0.68 mg, CCG-222740 concentration dissolved in methanol = 2 mg/ml). Solvents were removed from the round bottom flask (VWR, UK) using a rotary evaporator (Model R-100, BUCHI, UK) and a thin dry drug-loaded lipid film was obtained. Following the initial evaporation of the organic solvents and the thin film formation using the rotary evaporator, we continued the evaporation procedure at ‘full power’ for at least 30 more minutes to make sure that no traces of solvents were left. 1 ml of MilliQ-filtered water was added to hydrate the film. The mixture was sonicated for 5–10 min to form large unilamellar vesicles (LUVs) using a sonicator (Grant Instruments Ltd, UK). After completion of the sonication step, the resulting formulation was extruded through a 0.22 μm polycarbonate filter membrane (Merck Millipore, USA).

### Cell culture

Human conjunctival fibroblasts were grown from conjunctival tissues collected from glaucoma patients during glaucoma filtration surgery. Informed consent was obtained from all subjects. The fibroblasts were maintained in Dulbecco’s modified Eagle’s medium (DMEM, Invitrogen, UK) with 10% fetal calf bovine serum (FBS), 100 U/ml penicillin, 100 mg/ml streptomycin, and 2 nM l-glutamine, in tissue culture incubators with 5% CO_2_ and 95% humidity. Fibroblasts between passages 2–8 were used in all experiments. All experimental protocols were approved by the London-Dulwich Research Ethics Committee (REC 10/H0808/127) and the institutional approval committee at the University College London Institute of Ophthalmology.

### Size and zeta potential

Nanocarrier size and zeta potential were determined by dynamic light scattering (DLS) and laser Doppler anemometry, respectively, using a Nano ZS Zetasizer (Malvern Instruments, Malvern, UK) with the following specifications: automatic sampling time of 10 measurements/sample, refractive index 1.330, dielectric constant 78.5, viscosity 0.887 cP, and temperature of 25 °C. Zeta potential settings were calibrated against the standard (− 68 ± 6.8 mV). Triplicate measurements were performed for each sample and the results were analysed using the software provided by the manufacturer (DTS version 5.03).

### Transmission electron microscopy (TEM)

The nanocarrier CCG-222740 formulation was applied onto a 300-mesh copper grid coated with a Formvar/carbon support (Agar Scientific, Stansted, UK) and processed as previously described [22]. The sample was negatively stained with 1% phosphotungstic acid for 2–3 s, before blotting with filter paper and air-drying. Imaging was performed with a Philips CM120 BioTwin Transmission Electron Microscope and operated at an accelerating voltage of 120 kV. Images were captured using an AMT 5MP digital TEM camera (Deben UK Limited, UK).

### Drug release studies

Drug release studies were performed using an acrylic flow rig model consisting of a 200-μl cell to mimic the surgical bleb area in GFS [2]. The rig had both input and output tubes. The input tube was connected to a high precision multi-channel dispenser (ISMATEC pump, Switzerland) to pump MilliQ-filtered water at a flow rate of 2 μl/min to simulate the typical flow of aqueous humour in the eye. The experiments were performed at 37 °C to mimic the temperature of the subconjunctival space. The output tube was used for collection of samples at various time points. Aliquots from the release medium were collected in amber colour HPLC vials (2 ml, Agilent, UK) and stored at 4 °C until they were measured. The drug release experiments were performed in triplicates per condition and each experiment was repeated three times.

### High performance liquid chromatography (HPLC)

The drug concentrations were measured using the Agilent Technologies 1260 Infinity HPLC system (Agilent, UK) and detected by UV absorbance. A reverse phase Agilent Eclipse XDB-C18 column (5 μm, 4.6 × 150 mm) was used at 1 ml/min flow rate, injection volume of 10 μl, and UV detection wavelength of 254 nm. The mobile phase was acetonitrile: 0.1% trifluoroacetic acid at 65:35 proportions (volume/volume percentages). The drug estimation from the release medium was performed directly from the collected samples. The HPLC system was calibrated (R^2^ = 0.998) using known concentrations of CCG-222740 (2.5, 5, 10, 20, 30, 40, 50 μg/ml) by diluting CCG-222740 in methanol.

### Real-time quantitative PCR

Human conjunctival fibroblasts were plated at 1 × 10^5^ cells/well in 6-well plates (Falcon, Fisher Scientific). The next day, the fibroblasts were treated with 1.25, 2.5, 5 or 10 μM of nanocarrier CCG-222740 or empty liposomes in DMEM + 10% FCS for 24 h. The fibroblasts were then lysed for RNA extraction using the RNeasy mini kit (Qiagen, UK) according to the manufacturer’s instructions. RT-qPCR reactions were performed using a SensiFAST™ SYBR Hi-ROX One-Step master mix (Bioline, UK) on a QuantStudio5 Real-Time PCR system (ThermoFisher, UK). The qRT-PCR assay conditions were: stage 1, 45 °C for 20 min; stage 2, 95 °C for 3 min; stage 3, 95 °C for 10 s, then 60 °C for 25 min; repeated 40 times. The human Taqman gene expression assays were ACTA2 (Hs00426835_g1) and GAPDH (Hs02786624_g1) (ThermoFisher Scientific, UK). All mRNA values were normalised relative to that of GAPDH and triplicate experiments were performed for each condition.

### Cell proliferation assay

Human conjunctival fibroblasts were plated at 6.25 × 10^3^ cells/well in 96-well plates (Falcon, Fisher Scientific, UK), and treated with 1.25, 2.5, 5 or 10 μM of nanocarrier CCG-222740 or empty liposomes for 24 h. The medium was replaced by 100 μl fresh growth medium per well and 20 μl of the Cell Titer 96 Aqueous one solution (Promega, UK) were added to each well. The cells were incubated for 2 h and absorbance at 540 nm was measured using a FLUOstar Omega (BMG LABTECH, UK). Cell viability for each formulation was expressed as a percentage of the viability of control cells. Each experiment was carried out with six independent replicates for each condition.

### Rabbit glaucoma filtration surgery (GFS) model of scar tissue formation

All animal procedures were performed in accordance with the Association of Research in Vision and Ophthalmology (ARVO) Statement for the Use of Animals in Ophthalmic and Vision Research, and all animal experimental protocols were approved by the Home Office UK (PPL 70/8074). Eighteen female New Zealand white rabbits (1.5–2 kg, 10–12 weeks, Envigo, UK) underwent experimental GFS to the left eye under general anaesthesia [23, 24]. A superonasal fornix-based conjunctival flap was raised behind the limbus and a micro-vitreoretinal blade (20 gauge, 0.90 mm, Surgistar USA) was used to make a partial thickness scleral tunnel to the corneal stroma. A 22 gauge, 25 mm intravenous cannula was passed through the tunnel and the needle was removed once it was visible in the cornea. The cannula was advanced into the mid-pupillary area, trimmed at its scleral edge and fixed to the scleral surface using a 10-0 nylon suture (Alcon, USA). The conjunctival incision was closed using two interrupted 10-0 nylon sutures. In a randomised, prospective, single-masked observer study, the rabbits received either intraoperative 0.2 mg/ml mitomycin-C (MMC) [N = 6], or postoperative subconjunctival injections of 68 μg in 100 μl of nanocarrier CCG-222740 [N = 6] or empty liposomes [N = 6].

### Post-operative clinical examination

The animals were evaluated every 3 days by a single-masked blinded researcher. Bleb width and length were measured using calipers and intraocular pressures were measured using a tonovet (Icare, UK). The primary efficacy end-point was bleb survival as this is indicative of the long-term patency of the filtration pathway created during surgery. Bleb failure was defined as the appearance of a flat, scarred and vascularised bleb associated with a deep anterior chamber.

### Histologic analysis

The animals were sacrificed on day 30 and both eyes were enucleated. The eyes were fixed in formalin, embedded in paraffin, and sequential 4 μm tissue sections were cut. The sections were stained with hematoxylin and eosin (for cellularity and inflammatory cells), Gomori’s trichrome (for collagen), picrosirius red (for degree of fibrosis), and alpha-smooth muscle actin (αSMA) using a primary monoclonal mouse anti-human αSMA antibody (Clone 1A4; Dako, High Wycombe, UK) and a biotinylated secondary antibody (rabbit anti-mouse; Dako). All the left operated eyes were compared to the right non-operated eyes that were used as controls for normal conjunctival tissue.

### Toxicity and pharmacokinetics

All animals were evaluated for signs of local or systemic toxicity during the study. Conjunctival vascularity was graded as 0, avascular; 1, normal; 2, hyperaemic; 3, very hyperaemic. Anterior chamber inflammation was graded as 0, quiet; 1, cells; 2, fibrin; 3, hypopyon. At day 30, 100 µl of aqueous and 1 ml of vitreous were sampled from the rabbit eyes receiving nanocarrier CCG-222740 or empty liposomes. The levels of CCG-222740 were measured in the aqueous and vitreous samples using the HPLC method as described above.

### Statistical analysis

All graphs display mean and standard error of the mean (SEM). Statistical analysis was performed using the Student’s *t* test to calculate statistically significant differences and *p* values. Survival analysis for bleb was performed using the Kaplan–Meier log rank test. Statistically significant differences were expressed as *p < 0.05; **p < 0.01; ***p < 0.001.

## Results

### Development and biophysical characterisation of nanocarrier CCG-222740 formulation

**Fig. 1:**
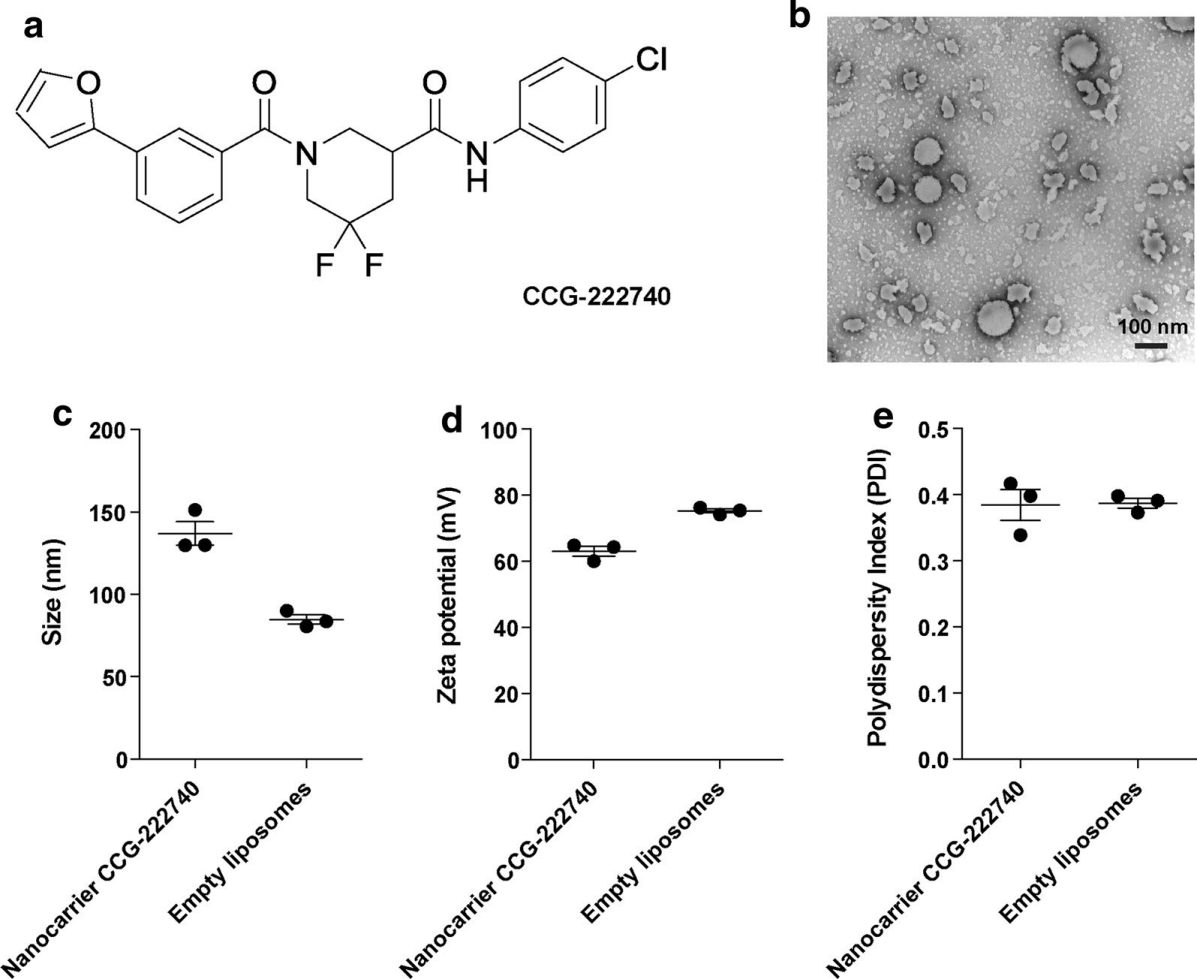
Molecular structure of an MRTF/SRF inhibitor **a** CCG-222740. **b** Negative staining transmission electron microscopy of the nanocarrier CCG-222740 formulation. **c** Size; **d** Zeta potential; and **e** Polydispersity index of the nanocarrier CCG-222740 formulation and empty liposomes were measured using a Nano ZS Zetasizer. Results represent mean ± SEM for triplicate measurements

Figure 1a shows the molecular structure of the MRTF/SRF inhibitor CCG-222740 (MW 444.9 g/mol). The nanocarrier CCG-222740 formulation was spherical particles as shown by negative-staining transmission electron microscopy (Fig. 1b). Nanocarrier CCG-222740 had a mean size of 137.0 ± 7.1 nm compared to 84.8 ± 2.8 nm for empty liposomes (Fig. 1c). Both nanocarrier CCG-222740 and empty liposomes were strongly cationic with a mean charge of +63.0 ± 1.5 mV and +75.2 ± 0.6 mV, respectively (Fig. 1d). Nanocarrier CCG-222740 and empty liposomes had relatively low polydispersity indices (PDI) of 0.39 ± 0.02 and 0.39 ± 0.01, respectively, indicating a narrow particle size distribution (Fig. 1e).

### Effect of nanocarrier CCG-222740 on *ACTA2* gene expression and cell viability in human conjunctival fibroblasts

**Fig. 2:**
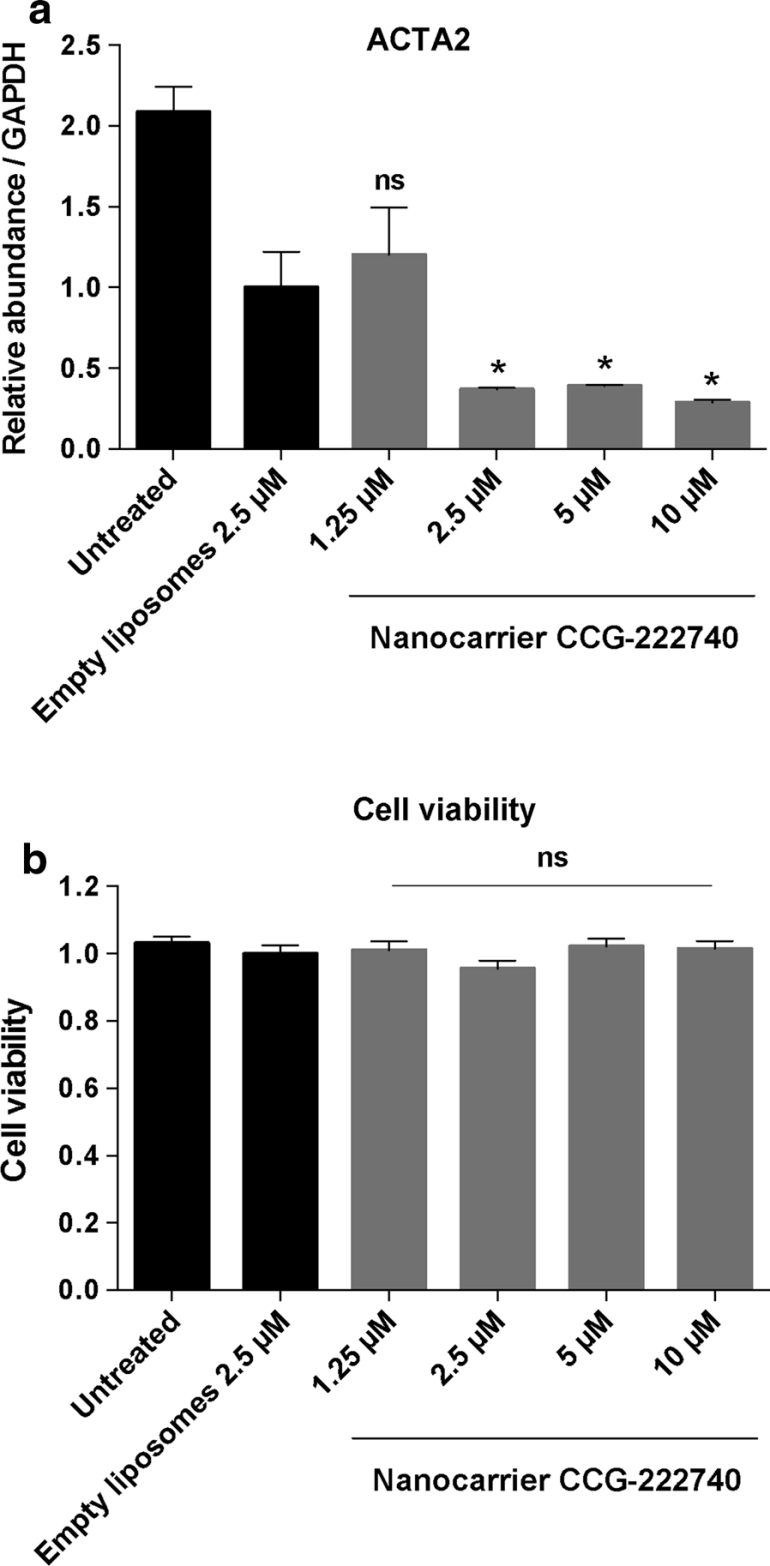
Human conjunctival fibroblasts were treated with different concentrations of nanocarrier CCG-222740 or empty liposomes for 24 h. **a** *ACTA2* gene expression was measured by real-time quantitative PCR. Results represent mean ± SEM for triplicate experiments. *P < 0.05. Comparisons shown are between cells treated with different concentrations of nanocarrier CCG-222740 or empty liposomes. **b** Effect on cell viability was measured using CellTiter 96 Aqueous one solution assay. Results represent mean ± SEM for six replicates per condition. The comparisons of cell viabilities shown are between cells treated with different concentrations of nanocarrier CCG-222740 or empty liposomes

*ACTA2*, coding for alpha-smooth muscle actin, is a direct target gene of the MRTF/SRF pathway that is classically linked to fibrosis [12]. Our results show that nanocarrier CCG-222740 formulation significantly decreased *ACTA2* gene expression in human conjunctival fibroblasts by 63.0% (*p* = 0.0468), 61.0% (*p* = 0.0257) and 71.2% (*p* = 0.0327) at 2.5, 5 and 10 μM concentrations, respectively, compared to empty liposomes (Fig. 2a). There was a vehicle effect with empty liposomes also causing a decrease in the *ACTA2* gene expression compared to untreated cells. This is a non-specific effect which has been observed by others in siRNA [25, 26] or drug-loaded liposomes [27]. Most likely this effect would not be seen if repeat transfections were performed. There were no significant differences in the *ACTA2* gene between empty liposomes and 1.25 μM nanocarrier CCG-222740 (Fig. 2a).

As 24 h of treatment were sufficient to determine a significant decrease in *ACTA2* gene expression in vitro in human conjunctival fibroblasts, we have thus measured cell viability 24 h post-transfection similar to previous studies [20]. We next tested the toxicity of nanocarrier CCG-222740 on human conjunctival fibroblasts. Different concentrations of nanocarrier CCG-222740 did not have any significant effect on cell viability compared to empty liposomes and untreated cells (Fig. 2b).

### Nanocarrier CCG-222740 achieves a sustained drug release as measured by high performance liquid chromatography

**Fig. 3:**
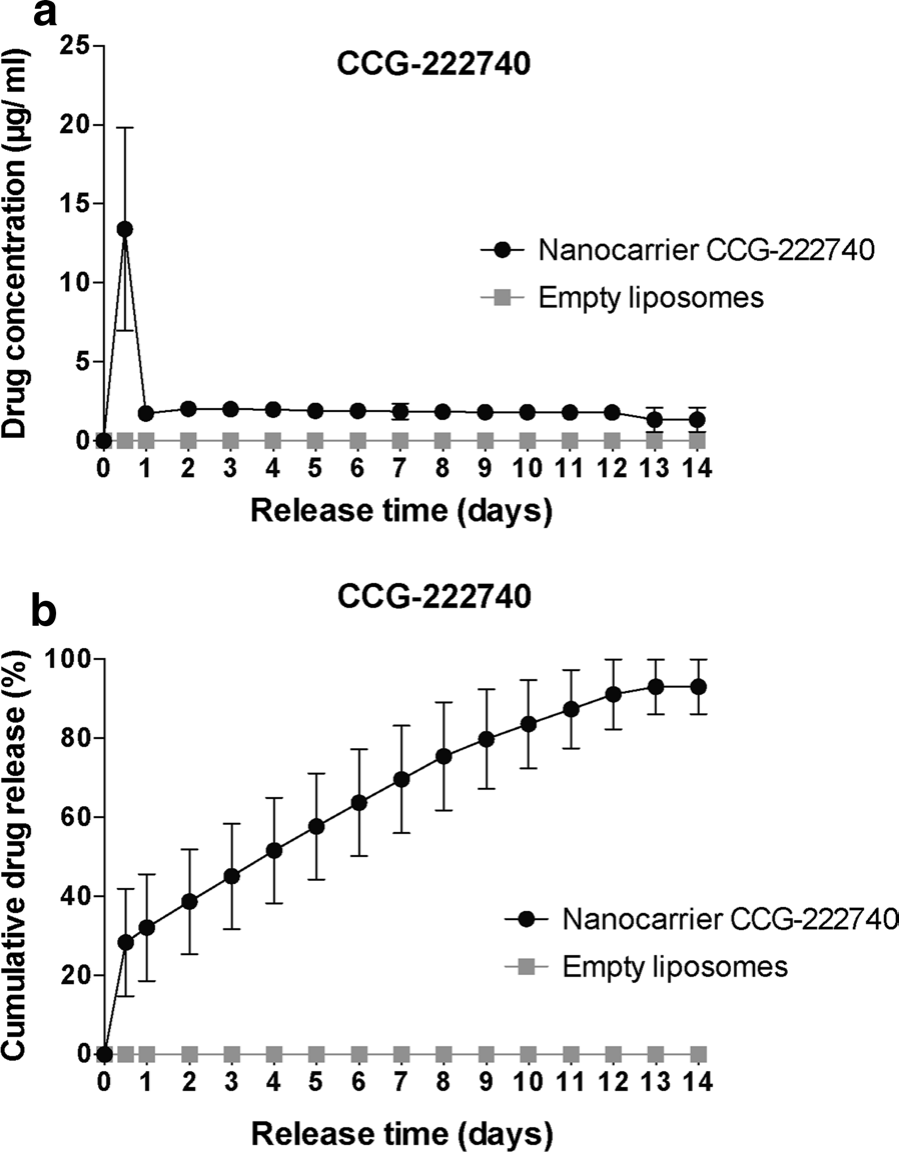
Drug release studies of nanocarrier CCG-222740. **a** Concentration curve of nanocarrier CCG-222740 and empty liposomes over 14 days. **b** Cumulative drug release of nanocarrier CCG-222740 and empty liposomes over 14 days. Results represent mean ± SEM for three independent experiments

We used a customised 200-μl cell with an open flow system for our drug release experiments as it mimics the conditions of the subconjunctival space, namely flow of aqueous humour and temperature, and allows collection of the released media at different time points [2]. We first calculated a calibration curve (y = 26.399x − 34.826; R^2^ = 0.99788) for CCG-222740 and used it to measure the drug concentrations in the released media using HPLC. The drug release concentration peaked at 13.4 ± 6.4 μg/ml at 0.5 day and stabilised at approximately 2 μg/ml from day 1 thereafter (Fig. 3a).

We used a flow rate of 2 μl per minute to simulate the typical flow of aqueous humour in the eye, so that 2.88 ml were collected over 24 h. The mean amount of drug released was 19.3 μg (13.4 μg/ml × 1.44 ml) at 0.5 day and approximately 5.8 μg daily (2 μg/ml × 2.88 ml) from day 1 thereafter. Out of the total of 68 μg of drug used, the nanocarrier CCG-222740 formulation led to a sustained cumulative drug release of 28.4 ± 13.6%, 32.1 ± 13.5%, 45.1 ± 13.3%, 63.7 ± 13.5%, 79.8 ± 12.6%, 91.1 ± 8.8% and 93.1 ± 6.9% at 0.5, 1, 3, 6, 9, 12 and 14 days, respectively (Fig. 3b).

### In vivo administration of nanocarrier CCG-222740 increases the long-term success of surgery in a rabbit model of GFS

**Fig. 4:**
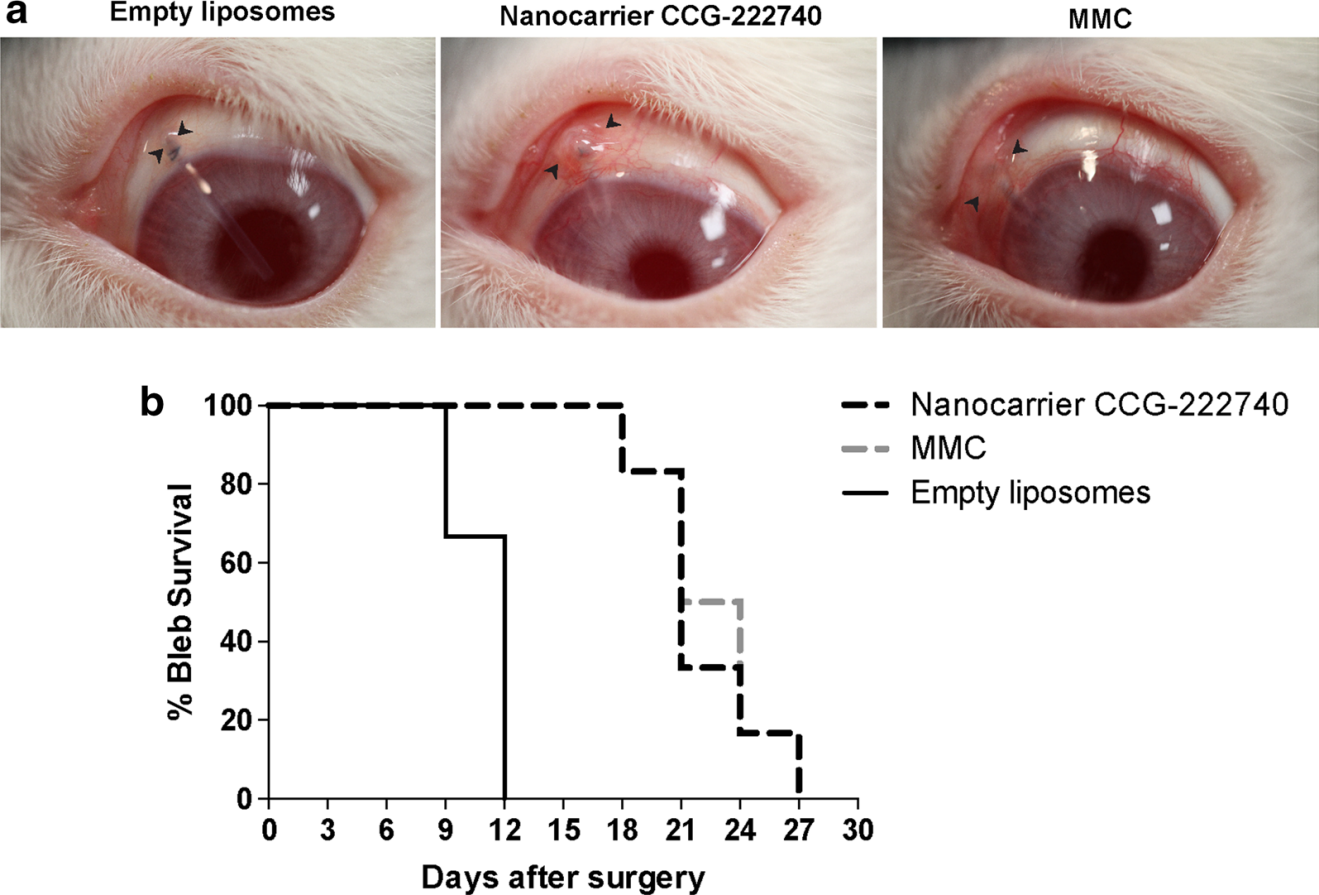
Effect of nanocarrier CCG-222740 on a rabbit model of glaucoma filtration surgery. **a** Morphology of blebs after surgery and treatment with nanocarrier CCG-222740, empty liposomes or mitomycin-C (MMC) at day 14. Arrows indicate bleb edges. **b** Kaplan–Meier graph comparing the bleb survival between nanocarrier CCG-222740 [N = 6], empty liposomes [N = 6] and MMC [N = 6]

We used an established and clinically validated rabbit model of experimental GFS to investigate the effects of nanocarrier CCG-222740 on wound healing in the conjunctiva [20, 23, 24]. Subconjunctival scarring after glaucoma filtration surgery is one of the most aggressive models of scar tissue formation, and failure of surgery is due to excessive scarring. A bleb arises when a filtration cannula is inserted during the surgery and drains aqueous fluid from the anterior chamber of the eye to under the conjunctiva. The primary efficacy endpoint of the study was bleb survival as this is indicative of the long-term opening of the filtration pathway created during the surgery. Bleb survival doubled from 11.0 ± 0.6 days for empty liposomes to 22.0 ± 1.3 days for nanocarrier CCG-222740 (*p* = 0.001) (Fig. 4a and b). We also compared the bleb survival between the standard dose of mitomycin-C (MMC, 0.2 mg/ml) and nanocarrier CCG-222740 (inhibitor concentration, 0.68 mg/ml). The surgical outcome for nanocarrier CCG-222740 was similar to that of MMC, which had a bleb survival of 22.5 ± 1.3 days.

### Nanocarrier CCG-222740 decreases conjunctival scarring and does not cause any local or systemic side effects

**Fig. 5:**
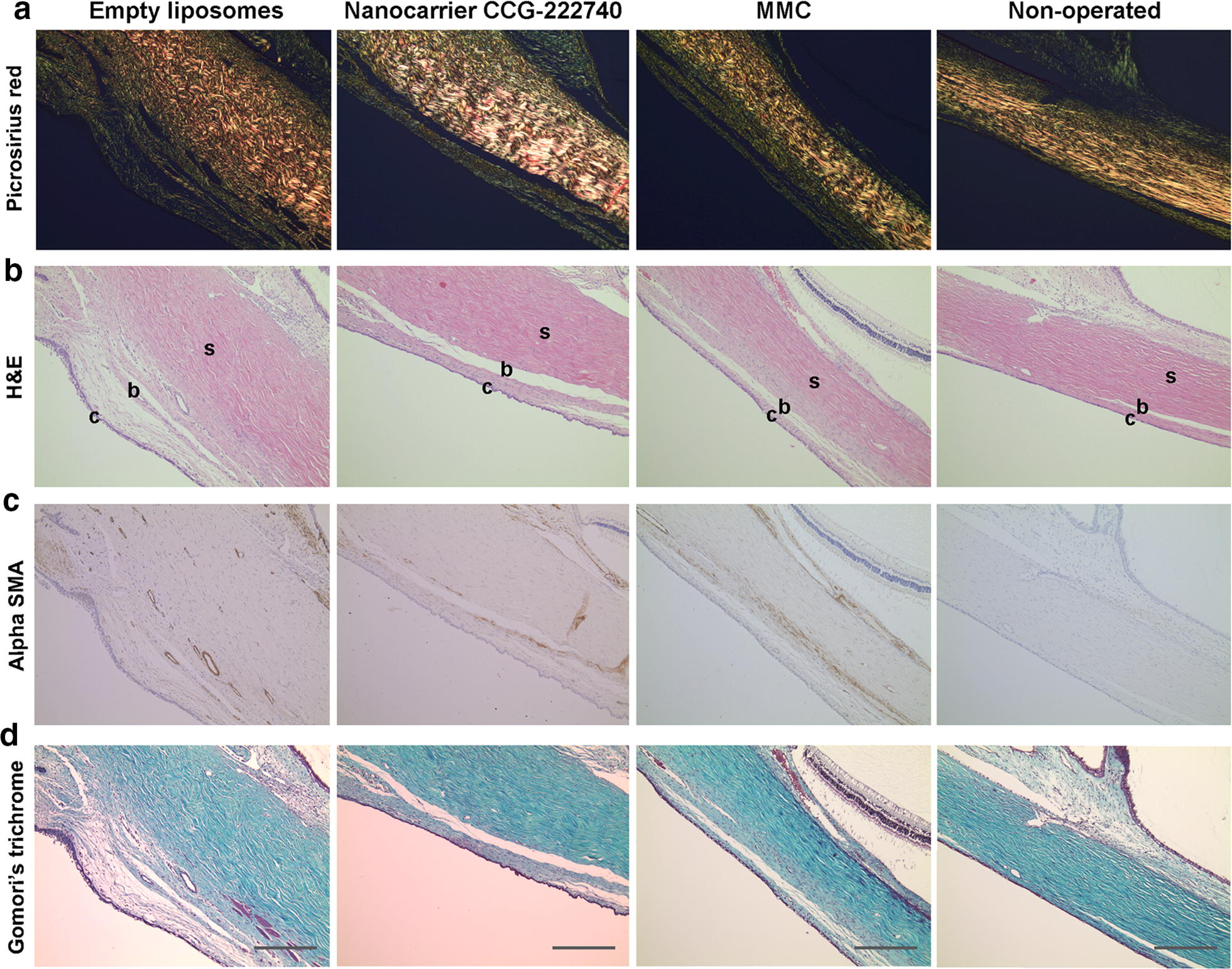
Histology of rabbit conjunctival tissues. The left operated eyes were compared to the right non-operated eyes that were used as controls for normal conjunctival tissue. Scale bar = 100 μm; c, conjunctiva, b, subconjunctival space, s, sclera. **a** Picrosirius red; **b** H&E; **c** Alpha-smooth muscle actin (αSMA); **d** Gomori’s trichrome stain

We evaluated the histologic differences in the conjunctiva after local delivery of nanocarrier CCG-222740. Our results show that nanocarrier CCG-222740 significantly decreased the scar tissue formation in the rabbit conjunctiva compared to empty liposomes (Fig. 5a). Total cellularity was also significantly increased in the empty liposomes group compared to the nanocarrier CCG-222740 group (Fig. 5b). In addition, nanocarrier CCG-222740 significantly decreased the expression of alpha-smooth muscle actin (αSMA) by cells, suggesting the presence of fewer myofibroblasts (Fig. 5c). The presence of newly laid collagen was also present to a greater degree in the vehicle control group compared to the nanocarrier CCG-222740 group (Fig. 5d).

**Fig. 6:**
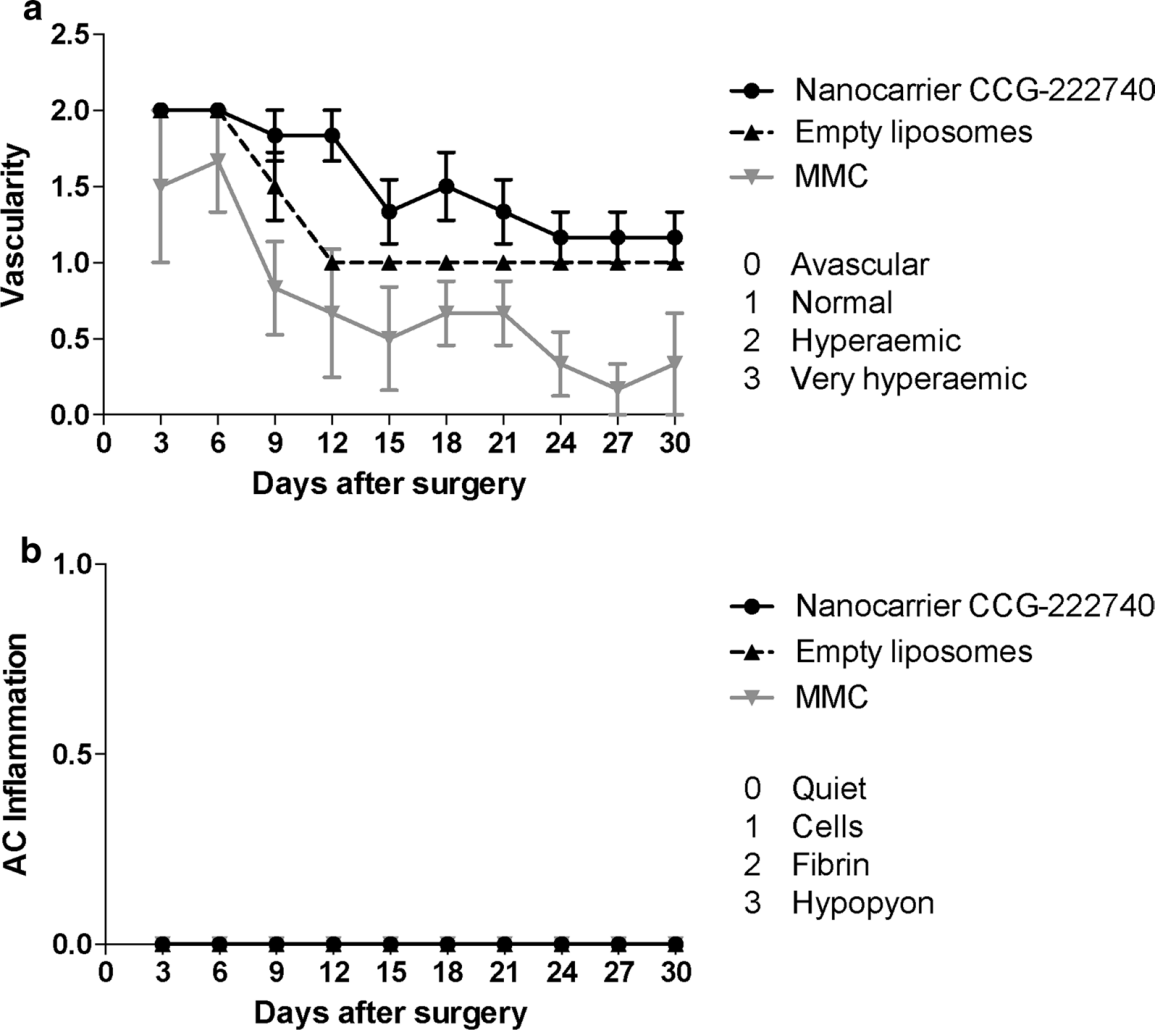
Nanocarrier CCG-222740 does not cause any local toxicity. **a** Conjunctival vascularity was graded as 0, avascular; 1, normal; 2, hyperaemic; 3, very hyperaemic. **b** Anterior chamber inflammation was graded as 0, quiet; 1, cells; 2, fibrin; 3, hypopyon

Local tissue reaction to treatment was further assessed by conjunctival vascularity. MMC treatment led to thin avascular blebs with a vascularity grade of 0.7 ± 0.2 (SEM) at day 30 compared to 1.3 ± 0.1 for empty liposomes (*p* = 0.022) (Fig. 6a). At day 30, there were no statistically significant differences in the vascularity grade between the nanocarrier CCG-222740-treated group (1.5 ± 0.1) and the empty liposomes-treated group (1.3 ± 0.1, *p* = 0.121). There were also no signs of corneal or conjunctival toxicities or anterior chamber inflammation (inflammation grade = 0) noted after local delivery of the nanocarrier CCG-222740 formulation (Fig. 6b).

In addition, the rabbits were carefully monitored throughout the study and none of them showed any signs of systemic toxicity after local delivery of the nanocarrier CCG-222740 formulation to the conjunctiva. We also tested the concentrations of CCG-222740 at day 30 in the aqueous and vitreous of the rabbits using HPLC. Levels of CCG-222740 were very low or undetectable in all the aqueous and vitreous samples.

## Discussion

Therapeutic nanomedicine is an area of great research interest in glaucoma [28] and our study provides proof-of-concept that small molecule inhibitors can be formulated into nanocarrier liposomal formulations for sustained drug delivery to the conjunctiva. The subconjunctival space is an area with constant flow of aqueous fluid and drugs are rapidly cleared from the subconjunctival space after GFS. Development of sustained release drug formulations with better pharmacokinetics and longer duration of action is thus crucial for successful drug delivery to the conjunctiva after GFS.

Large unilamellar vesicles (LUVs) and multilamellar vesicles (MLVs) can significantly increase the bioavailability and efficacy of drugs in the eye. MLVs containing acetazolamide were more efficient than an acetazolamide solution in lowering the intraocular pressure (IOP) [29]. LUVs containing latanaprost also maintained the IOP-lowering effect of latanaprost for up to 50 days in a rabbit model [30]. In addition, liposomal ganciclovir had an ocular bioavailability that was 1.7-fold higher than that of a ganciclovir solution [31]. Charge also plays an important role in sustained drug delivery and positively charged or neutral liposomes tend to have a more prolonged effect than negatively charged ones [29]. In our study, the liposomes were strongly cationic and gradually released the MRTF/SRF inhibitor over a sustained period of 14 days. Several administrations of the nanocarrier CCG-222740 formulation, for example every 2 weeks, would achieve a more prolonged release of the drug to prevent conjunctival fibrosis in the post-operative period after GFS.

Different liposomes and drug: liposome ratios are currently being tested for different types of drugs. In this study, we used inhibitor: DOTMA: DOPC in a 1:1:1 molar ratio and we incorporated a large amount of inhibitor in the liposomes. Latanaprost: di-palmitoyl-phosphatidyl-choline formulations at a molar ratio of 1:10 also had good physical stability and release, and demonstrated sustained release of latanoprost in vitro and in a rabbit model [30, 32]. MLVs composed of phosphatidylcholine: cholesterol: stearylamine in a 7:4:1 ratio also effectively packaged acetazolamide, which was adjusted at a concentration of 1%, and demonstrated maximal lowering effect of the intraocular pressure [29].

Using a high-throughput screen with an MRTF/SRF reporter gene that selectively responds to RhoA-induced gene transcription [33], CCG-1423 was identified as a first generation inhibitor of RhoA-stimulated MRTF/SRF gene transcription [34]. A second generation inhibitor, CCG-203971, was optimised that is of equal efficacy but less cytotoxic than CCG-1423 [35, 36]. Another second generation MRTF/SRF pathway inhibitor, CCG-222740, has also been developed and is more potent and metabolically stable than CCG-203971 [20, 37]. However, the molecular target of the MRTF/SRF inhibitors has not yet definitively been established. A previous study on the MRTF/SRF pathway inhibitor CCG-1423 reported that it increases nuclear G-actin and decreases MRTF-A in the nucleus through inhibition of MICAL-2, a member of the MICAL family of actin-binding mono-oxygenases [38]. Another study also reported that CCG-1423 acts directly on MRTF and other RPEL-family of proteins to prevent the nuclear import of MRTF-A [39].

Mitomycin-C is a toxic antimetabolite and exerts its anti-scarring effects by arresting the cell cycle and causing widespread apoptosis of fibroblasts in the conjunctiva [40]. Vision-threatening side effects have been associated with the use of mitomycin-C, namely hypotonous maculopathy [41], severe infection [42], and corneal melting and perforation [43]. Our previous study showed that repeated subconjunctival injections of CCG-222740 did not cause any detectable local toxicity with an intact and healthy-looking conjunctival epithelium that was similar to the non-operated eyes, and with normal Ki67 positive cells (marker of cell proliferation) in the conjunctival epithelium [20]. Similarly, our current study showed that the nanocarrier liposomal formulation of CCG-222740 was not associated with any signs of corneal or conjunctival toxicities or anterior chamber inflammation. MMC treatment led to thin avascular blebs but there were no abnormalities in conjunctival vascularity noted in the nanocarrier CCG-222740-treated group. Other studies have also reported that nanosized liposomal formulations of drugs do not lead to significant toxicity and inflammation in the eye [21, 30].

## Conclusion

Our study provides proof-of-concept that a nanocarrier-based formulation achieved a sustained release of an MRTF/SRF inhibitor over time and prevented conjunctival fibrosis in a rabbit model of experimental GFS. The nanocarrier formulation was also safe to use and did not cause any ocular or systemic adverse effects.

## Abbreviations

GFS: glaucoma filtration surgery
MRTF: myocardin-related transcription factor
SRF: serum response factor
HPLC: high performance liquid chromatography
DLS: dynamic light scattering
TEM: transmission electron microscopy
PCR: polymerase chain reaction
αSMA: alpha-smooth muscle actin
IOP: intraocular pressure
MMC: mitomycin-C
LUVs: large unilamellar vesicles
MLVs: multilamellar vesicles
DOTMA: 1,2-di-*O*-octadecenyl-3-trimethylammonium propane
DOPC: 1,2-dioleoyl-*sn*-glycero-3-phosphocholine
SEM: standard error of the mean

## References

[1] Ashley GW, Henise J, Reid R, Santi DV (2013). Hydrogel drug delivery system with predictable and tunable drug release and degradation rates. Proc Natl Acad Sci USA..

[2] Parkinson G, Gaisford S, Ru Q, Lockwood A, Khalili A, Sheridan R (2012). Characterisation of Ilomastat for prolonged ocular drug release. AAPS Pharm Sci Tech.

[3] Ru Q, Fadda HM, Li C, Paul D, Khaw PT, Brocchini S (2013). Molecular dynamic simulations of ocular tablet dissolution. J Chem Inf Model.

[4] Blaker JJ, Knowles JC, Day RM (2008). Novel fabrication techniques to produce microspheres by thermally induced phase separation for tissue engineering and drug delivery. Acta Biomater.

[5] Liu Y-C, Peng Y, Lwin NC, Wong TT, Venkatraman SS, Mehta JS (2013). Optimization of subconjunctival biodegradable microfilms for sustained drug delivery to the anterior segment in a small animal model. Invest Ophthalmol Vis Sci.

[6] Rodríguez-Agirretxe I, Vega SC, Rezola R, Vecino E, Mendicute J, Suarez-Cortes T (2013). The PLGA implant as an antimitotic delivery system after experimental trabeculectomy. Invest Ophthalmol Vis Sci.

[7] Tham YC, Li X, Wong TY, Quigley HA, Aung T, Cheng CY (2014). Global prevalence of glaucoma and projections of glaucoma burden through 2040: a systematic review and meta-analysis. Ophthalmology.

[8] Yu-Wai-Man C, Tagalakis AD, Meng JH, Bouremel Y, Lee RM, Virasami A (2017). Genotype-phenotype associations of IL-6 and PRG4 with conjunctival fibrosis after glaucoma surgery. JAMA Ophthalmol..

[9] Treisman R (1986). Identification of a protein-binding site that mediates transcriptional response of the c-fos gene to serum factors. Cell.

[10] Norman C, Runswick M, Pollock R, Treisman R (1988). Isolation and properties of cDNA clones encoding SRF, a transcription factor that binds to the c-fos serum response element. Cell.

[11] Treisman R (1992). The serum response element. Trends Biochem Sci.

[12] Esnault C, Stewart A, Gualdrini F, East P, Horswell S, Matthews N (2014). Rho–actin signaling to the MRTF coactivators dominates the immediate transcriptional response to serum in fibroblasts. Genes Dev.

[13] Olson EN, Nordheim A (2010). Linking actin dynamics and gene transcription to drive cellular motile functions. Nat Rev Mol Cell Biol.

[14] Haak AJ, Tsou PS, Amin MA, Ruth JH, Campbell P, Fox DA (2014). Targeting the myofibroblast genetic switch: inhibitors of MRTF/SRF-regulated gene transcription prevent fibrosis in a murine model of skin injury. J Pharmacol Exp Ther.

[15] Minami T, Kuwahara K, Nakagawa Y, Takaoka M, Kinoshita H, Nakao K (2012). Reciprocal expression of MRTF-A and myocardin is crucial for pathological vascular remodelling in mice. EMBO J.

[16] Sisson TH, Ajayi IO, Subbotina N, Dodi AE, Rodansky ES, Chibucos LN (2015). Inhibition of myocardin-related transcription factor/serum response factor signaling decreases lung fibrosis and promotes mesenchymal cell apoptosis. Am J Pathol.

[17] Yu-Wai-Man C, Tagalakis AD, Manunta MD, Hart SL, Khaw PT (2016). Receptor-targeted liposome-peptide-siRNA nanoparticles represent an efficient delivery system for MRTF silencing in conjunctival fibrosis. Sci Rep..

[18] Vartiainen MK, Guettler S, Larijani B, Treisman R (2007). Nuclear actin regulates dynamic subcellular localization and activity of the SRF cofactor MAL. Science.

[19] Miralles F, Posern G, Zaromytidou AI, Treisman R (2003). Actin dynamics control SRF activity by regulation of its coactivator MAL. Cell.

[20] Yu-Wai-Man C, Spencer-Dene B, Lee RMH, Hutchings K, Lisabeth EM, Treisman R (2017). Local delivery of novel MRTF/SRF inhibitors prevents scar tissue formation in a preclinical model of fibrosis. Sci Rep..

[21] Hironaka K, Inokuchi Y, Tozuka Y, Shimazawa M, Hara H, Takeuchi H (2009). Design and evaluation of a liposomal delivery system targeting the posterior segment of the eye. J Control Rel.

[22] Tagalakis AD, Maeshima R, Yu-Wai-Man C, Meng J, Syed F, Wu LP (2017). Peptide and nucleic acid-directed self-assembly of cationic nanovehicles through giant unilamellar vesicle modification: targetable nanocomplexes for in vivo nucleic acid delivery. Acta Biomater.

[23] Cordeiro MF, Gay J, Khaw PT (1999). Human anti-transforming growth factor-beta2 antibody: a new glaucoma anti-scarring agent. Invest Ophthalmol Vis Sci.

[24] Wong TTL, Mead AL, Khaw PT (2003). Matrix metalloproteinase inhibition modulates postoperative scarring after experimental glaucoma filtration surgery. Invest Ophthalmol Vis Sci.

[25] Tagalakis AD, Lee DHD, Bienemann AS, Zhou H, Munye MM, Saraiva L (2014). Multifunctional, self-assembling anionic peptide-lipid nanocomplexes for targeted siRNA delivery. Biomaterials.

[26] McCaskill J, Singhania R, Burgess M, Allavena R, Wu S, Blumenthal A (2013). Efficient, biodistribution and gene silencing in the lung epithelium via intravenous liposomal delivery of siRNA. Mol Ther Nucleic Acids..

[27] Deng W, Chen W, Clement S, Guller A, Zhao Z, Engel A (2018). Controlled gene and drug release from a liposomal delivery platform triggered by X-ray radiation. Nat Commun..

[28] Yu-Wai-Man C, Khaw PT (2015). Developing novel anti-fibrotic therapeutics to modulate post-surgical wound healing in glaucoma: big potential for small molecules. Expert Rev Ophthalmol..

[29] Hathout RM, Mansour S, Mortada ND, Guinedi AS (2007). Liposomes as an ocular delivery system for acetazolamide: in vitro and in vivo studies. AAPS Pharm Sci Tech.

[30] Natarajan JV, Chattopadhyay S, Ang M, Darwitan A, Foo S, Zhen M (2011). Sustained release of an anti-glaucoma drug: demonstration of efficacy of a liposomal formulation in the rabbit eye. PLoS ONE.

[31] Shen Y, Tu J (2007). Preparation and ocular pharmacokinetics of ganciclovir liposomes. AAPS J.

[32] Natarajan JV, Ang M, Darwitan A, Chattopadhyay S, Wong TT, Venkatraman SS (2012). Nanomedicine for glaucoma: liposomes provide sustained release of latanoprost in the eye. Int J Nanomed..

[33] Suzuki N, Nakamura S, Mano H, Kozasa T (2003). Ga12 activates Rho GTPase through tyrosine-phosphorylated leukemia-associated RhoGEF. Proc Natl Acad Sci USA..

[34] Evelyn CR, Wade SM, Wang Q, Wu M, Iniguez-Lluhı JA, Merajver SD (2007). CCG-1423: a small-molecule inhibitor of RhoA transcriptional signaling. Mol Cancer Ther.

[35] Bell JL, Haak AJ, Wade SM, Kirchhoff PD, Neubig RR, Larsen SD (2013). Optimization of novel nipecotic bis(amide) inhibitors of the Rho/MKL1/SRF transcriptional pathway as potential anti-metastasis agents. Bioorg Med Chem Lett.

[36] Evelyn CR, Bell JL, Ryu JG, Wade SM, Kocab A, Harzdorf NL (2010). Design, synthesis and prostate cancer cell-based studies of analogs of the Rho/MKL1 transcriptional pathway inhibitor, CCG-1423. Bioorg Med Chem Lett.

[37] Hutchings KM, Lisabeth EM, Rajeswaran W, Wilson MW, Sorenson RJ, Campbell PL (2017). Pharmacokinetic optimitzation of CCG-203971: novel inhibitors of the Rho/MRTF/SRF transcriptional pathway as potential antifibrotic therapeutics for systemic scleroderma. Bioorg Med Chem Lett.

[38] Lundquist MR, Storaska AJ, Liu TC, Larsen SD, Evans T, Neubig RR (2014). Redox modification of nuclear actin by MICAL-2 regulates SRF signaling. Cell.

[39] Hayashi K, Watanabe B, Nakagawa Y, Minami S, Morita T (2014). RPEL proteins are the molecular targets for CCG-1423, an inhibitor of Rho signaling. PLoS ONE.

[40] Crowston JG, Akbar AN, Constable PH, Occleston NL, Daniels JT, Khaw PT (1998). Antimetabolites-induced apoptosis in Tenon’s capsule fibroblasts. Invest Ophthalmol Vis Sci.

[41] Jampel HD, Pasquale LR, Dibernardo C (1992). Hypotony maculopathy following trabeculectomy with mitomycin C. Arch Ophthalmol.

[42] Parrish R, Minckler D (1996). “Late endophthalmitis”: filtering surgery time bomb?. Ophthalmology.

[43] Dougherty PJ, Hardten DR, Lindstrom RL (1996). Corneoscleral melt after pterygium surgery using a single intraoperative application of mitomycin-C. Cornea.

